# Metabolic modulation of Ewing sarcoma cells inhibits tumor growth and stem cell properties

**DOI:** 10.18632/oncotarget.20467

**Published:** 2017-08-24

**Authors:** Atreyi Dasgupta, Matteo Trucco, Nino Rainusso, Ronald J. Bernardi, Ryan Shuck, Lyazat Kurenbekova, David M. Loeb, Jason T. Yustein

**Affiliations:** ^1^ The Faris D. Virani Ewing Sarcoma Center at The Texas Children's Cancer and Hematology Centers, Department of Pediatrics, Baylor College of Medicine, Houston, TX 77030, USA; ^2^ Sylvester Comprehensive Cancer Center, Department of Pediatrics, Hematology-Oncology, University of Miami-Miller School of Medicine, Miami, FL 33136, USA; ^3^ Sydney Kimmel Comprehensive Cancer Center at Johns Hopkins Hospital, Baltimore, MD 21231, USA; ^4^ Integrative Molecular and Biological Sciences Program, Baylor College of Medicine, Houston, TX 77030, USA; ^5^ Department of Molecular and Cellular Biology, Baylor College of Medicine, Houston, TX 77030, USA

**Keywords:** metabolism, cancer stem cells, Ewing sarcoma, 2DG, metformin

## Abstract

Ewing sarcoma (EWS) is a highly aggressive and metabolically active malignant tumor. Metabolic activity can broadly be characterized by features of glycolytic activity and oxidative phosphorylation. We have further characterized metabolic features of EWS cells to identify potential therapeutic targets. EWS cells had significantly more glycolytic activity compared to their non-malignant counterparts. Thus, metabolic inhibitors of glycolysis such as 2-deoxy-D-glucose (2DG) and of the mitochondrial respiratory pathway, such as metformin, were evaluated as potential therapeutic agents against a panel of EWS cell lines *in vitro*. Results indicate that 2DG alone or in combination with metformin was effective at inducing cell death in EWS cell lines. The predominant mechanism of cell death appears to be through stimulating apoptosis leading into necrosis with concomitant activation of AMPK-α. Furthermore, we demonstrate that the use of metabolic modulators can target putative EWS stem cells, both *in vitro* and *in vivo*, and potentially overcome chemotherapeutic resistance in EWS. Based on these data, clinical strategies using drugs targeting tumor cell metabolism present a viable therapeutic modality against EWS.

## INTRODUCTION

Ewing Sarcoma (EWS) is the second most common pediatric malignant bone tumor, accounting for 2% of all childhood cancers [1]. Ewing sarcoma develops not only in osseous sites (85%) but also in extra-skeletal soft tissues [2–4]. At diagnosis, approximately 25-30% of patients with EWS have metastatic disease [5]. For patients with localized disease, implementation of intensive multi-drug systemic chemotherapy regimen, along with surgery and/or radiotherapy, has led to survival rates of approximately 70%. However, patients with metastatic or recurrent disease have a dismal prognosis even after undergoing intensive, multi-modality treatment with long-term survival rates of approximately 20-30% [6, 7]. The identification of key molecular pathways or tumor cell subpopulations involved in the processes of metastasis and therapeutic resistance is critical to develop novel therapeutic approaches for patients with recurrent or metastatic ES.

Current research and therapeutic efforts have focused on identifying and targeting specific pathways and genes that would have a crucial role in the development of therapeutic resistance. About 95% of EWS family tumors contain a translocation between the EWS gene on chromosome 11 and the ETS family genes (FLI1 or ERG) on chromosome 22 [8]. The EWS-FLI1 fusion protein acts as an aberrant transcription factor and through its target genes, can cause neoplastic transformation. Many of these target genes have been implicated in tumor growth and progression [9, 10]. Recent targeted therapies directed towards molecular aberrations of these pathways including angiogenesis via molecules such as EWS-FLI1 [11, 12] itself or through VEGFRs [13, 14], or the use of imatinib (Gleevec) [15] to target protein tyrosine kinases such as PDGFR and cKit, cell surface receptors such as GD2, IGF-1R, and other pathways involving mTOR [16], EGFR [17], PARP1 [18, 19] etc. have yielded disappointing results. In spite of all these investigations, there is very limited data regarding which specific pathway needs to be targeted in patients who might be harboring residual disease and the last new agent that was added to the standard regimen, was the cytotoxic chemotherapeutic agent, ifosfamide, more than 15 years ago [20].

The lack of improvement in outcomes using these strategies suggests that alternative approaches are essential to make any further progress. Additionally, it is necessary for future therapeutic modality to target not just the localized tumor cells, but also, residual tumor cells in the body, either in the form of circulating tumor cells or cancer stem-like cells. In recent years, an increasing body of evidence suggests that persistence of cancer stem cells (CSCs) can partially account for radio-resistance, chemo-resistance, and overall tumor invasion and recurrence in many cancers including sarcomas. [21–23]. To target this tumor cell population, specific CSC markers need to be identified. Studies performed in prostate, lung, brain, and breast cancers have demonstrated that tumor cells with high aldehyde dehydrogenase (ALDH) activity possess stem cell properties, including but not limiting to re-initiation of serial tumor implants, and developing into a heterogeneous population of cancer cells [24–28]. Previous studies have also shown that EWS contains an ALDH^high^ stem-like population that can confer resistance to chemotherapy [29].

In recent years, in a search for alternative cancer therapeutic modalities, targeting tumor cell metabolism has generated heightened interest. The preference for many tumors to utilize glycolysis for cellular energy production, even in the presence of oxygen, has been termed the Warburg effect [30]. Such cancer cell adaptation, although intuitively might not seem to be energy efficient, suggests a key role in tumor progression because of its overwhelming presence in the majority of metastatic tumors, and is now recognized as a hallmark of cancer [31, 32]. This tumor adaptive response appears to be present in EWS cells as well. The production of high levels of lactate, as a surrogate marker for the presence of glycolysis, can be observed in EWS even when these cells are grown under normoxic conditions, indicating that glycolysis is constitutively active in this tumor [33].

Due to these features, EWS cells may be particularly sensitive to glycolysis inhibitors and cellular energy depleting agents that may induce profound levels of cytotoxic and cytostatic effects, and in turn inhibit tumor progression.

To target glycolysis, we used the glucose analog and competitive inhibitor 2-deoxy-D-glucose (2DG). 2DG binds to glucose transporters and gets phosphorylated by hexokinase to 2DG-6-phosphate, which cannot be metabolized any further, thus blocking the glycolytic pathway [34, 35]. Several clinical and pre-clinical studies have demonstrated 2DG's inhibitory effect on chemo-resistant tumor cells and on cancer stem-like cells [36–38].

Metformin is a frontline therapy for type 2 diabetes. The safety and side effects of metformin is well documented since it has been prescribed to more than 120 million diabetic patients world-wide. Epidemiologic, retrospective, and laboratory studies strongly suggest that metformin has anti-tumor effects in a wide range of cancer [39–44]. Metformin's anti-tumor effect can be both direct and indirect. The drug directly targets the insulin like-growth factor (IGF) and insulin pathway, both of which are known to have tumor promoting effects, while indirectly, metformin has been reported to be an inhibitor of the mitochondrial complex I respiratory chain, inhibiting the tricarboxylic acid (TCA) cycle and oxidative phosphorylation (OXPHOS), increasing lactate production, and rendering the tumor cells energetically inefficient [45, 46].

Our studies characterized some key metabolic features of EWS cells and examined the effects of inhibiting glycolysis and OXPHOS separately and simultaneously in EWS cells. Furthermore, our results indicate that these classes of compounds can be used to overcome therapeutic resistance and inhibit cancer stem-like cells in EWS, providing evidence that metabolic modulation has therapeutic potential for treatment of EWS.

## RESULTS

### Ewing sarcoma cells exhibit high metabolic and glycolytic activity

**Figure 1:**
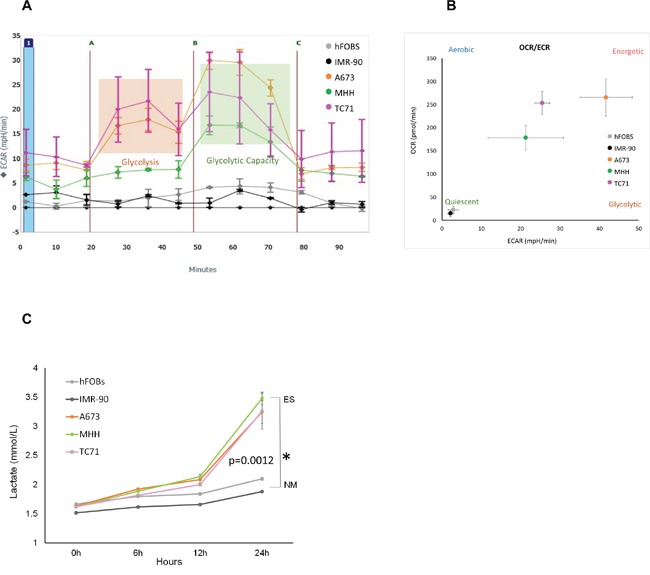
Metabolic profile of Ewing's sarcoma cells **(A)** ECAR rate was measured for EWS (A673, MHH, and TC71) and NM cells (hFOBS and IMR-90) in real-time using the Seahorse extracellular flux analyzer. A series of extracellular acidification rates (ECAR) were calculated showing basal glycolytic activity (orange block) and the maximum glycolytic capacity (green block). A, B, and C indicates injection of glucose, oligomycin and 2DG respectively. **(B)** OCR/ECAR ratio indicating dependence of cells on either mitochondrial respiration or glycolysis. Absolute values were obtained by seeding 30,000 cells/well. **(C)** Media at different time points from wells with 2×10^4^ cells were collected to determine extracellular lactate level, lactate measurements showed significant increase at 72 hours compared to the NM cells. *p <0.05 was regarded as significant and calculated by comparing the means of the two groups with unpaired t test. Data represents the mean of three wells ± standard deviation.

To characterize the bioenergetic profiles of the EWS cells, we did extracellular flux analysis on a panel of three established EWS cells using Seahorse XF24 Extracellular Flux Analyzer (Figure 1A). We quantified their glycolytic activity by measuring extracellular acidification rate (ECAR) as glycolytic cells produce more protons [47]. Relative contributions of different biochemical pathways were then measured by adding specific inhibitors. ECAR following the addition of glucose defines glycolysis and ECAR following oligomycin represents maximum glycolytic capacity. ECAR prior to the addition of glucose and following treatment with 2-DG represents acidification associated with non-glycolytic activity. Compared to the two non-malignant (NM) cells, all three EWS cells showed high ECAR, and that the EWS cells were very close to their maximum glycolytic capacity, even at resting state. The ratio between the oxygen consumption rate (OCR) and ECAR showed the EWS cells varied in their energy profile in terms of mitotic respiration, but overall, preferred aerobic glycolysis compared to the NM cells, which exhibited a less energetic profile (Figure 1B).

This was further confirmed by the lactate measurement (Figure 1C) from the culture media of three EWS cell lines, namely A673, MHH, TC71 and two non-malignant (NM) cell lines – hFOBS, and IMR-90. The lactate levels measured in the media of the EWS cell lines were higher than the NM cells from six hours onwards. At 24 hours, lactate produced by EWS cells were significantly higher (*p*= 0.0012), showing high glycolytic activity by these cancer cells compared to the NM cells.

### Metabolic inhibition with 2DG and metformin can modulate glycolytic activity in EWS cells

**Figure 2:**
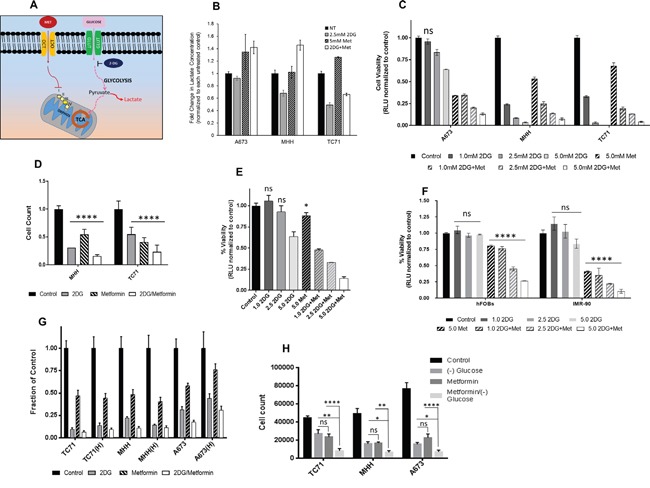
Targeting energy pathway by metabolic inhibition **(A)** Schematic showing the two main pathways of glucose metabolism. **(B)** Effect of metabolic inhibition on lactate formation. Media after no treatment and 24 hours treatment with 2DG (2.5 mM) or metformin (5 mM) or a combination, was collected from wells with 2×10^4^ cells to determine extracellular lactate level. Lactate measurements are shown as fold change over untreated control. **(C)** Effect of 2DG and Metformin on cell viability. EWS cells were treated for 3 days with varying concentrations of 2DG or metformin, alone or in combination. CellTiter-Glo was added and viability was measured at 72 hours. The results are expressed as relative fraction of viability compared with the corresponding untreated control group. Other than the indicated non-significant statistical difference (ns) all other treatment groups compared to the corresponding control was significantly different with *p* <0.0001. **(D)** Ewing sarcoma cells were treated for 3 days with 5 mM of 2DG, and 10 mM of metformin as single agents or in combination. Number of cells after treatment was quantified *in situ* with an image cytometer (Celigo). Data shown are means ± SD of 3 determinations. **(E)** PDX38 cell line, established from a EWS patient was used to see effect of metabolic inhibition on cell viability. Cells were treated for 3 days with indicated concentrations of 2DG and Metformin, alone or in combination. CellTiter-Glo was added and viability was measured at 72 hours. The results are expressed as relative fraction viability compared with the corresponding untreated control group. **(F)** Non-malignant cells, hFOBS and IMR-90 were treated for 3 days with indicated concentrations of 2DG, or metformin alone or in combination. **(G)** 2DG and metformin effects are independent of hypoxia. Cells were grown under normoxic conditions with 20% O_2_ or under 1% hypoxia for three days. Cells were left untreated or treated with either 2DG (5 mM), or metformin (10 mM) as single agents or in combination. Number of cells after treatment was quantified with *in situ* with an image cytometer (Celigo). **(H)** EWS cells either grown under normal culture condition with 25 mM glucose, or under glucose starved condition, were treated with 5 mM 2DG and 10 mM metformin either alone or in combination. Number of cells after treatment was quantified with *in situ* with an image cytometer (Celigo). Statistical significance of *p* <0.05 was calculated with two-way Anova with Dunnett's multiple correction (* <0.05, ** <0.01, *** <0.001, **** <0.0001) with ns indicating non-significant. All data, unless otherwise indicated had *p* <0.0001 by Dunnett's multiple comparison test, when compared to corresponding control.

We postulated that a combination of metformin and 2DG would significantly affect tumor cell proliferation and viability, as summarized in the schematic (Figure 2A). The two main glucose metabolism pathways involve a) oxidative phosphorylation in TCA cycle involving mitochondrial respiration complex I-IV and b) glycolysis. Metformin acts as the inhibitor of the former, while 2DG of the latter. Glucose transport across the cell membrane is mediated by the glucose transporters (*GLUT*) while organic cation transporters (*OCT*) actively transports metformin (*MET*) inside the cell. Metformin-induced mitochondrial respiration inhibition in EWS cells would increase lactate production, as cells would try to “rescue” energy production through aerobic glycolysis. On the other hand, treatment with 2DG should inhibit aerobic glycolysis, as can be observed by the decrease in lactate production and consequently inhibit tumor cell viability. Thus, the combination of metformin and 2DG would have the maximal effect in inhibiting the mechanisms of energy production in EWS cells. To test this hypothesis, we treated cells either singly or in combination with 2DG at 2.5 mM and metformin at 5 mM concentration for 24 hours (Figure 2B). From lactate measurement at the end of 24 hours (normalized to cell numbers), we observed that lactate production was increased for all cell lines under metformin treatment, whereas, with 2DG we observed a decrease in two of the three cell lines, namely MHH and TC71.

### 2DG and metformin can inhibit EWS tumor cell viability

To see if modulating the cell's metabolism can result in inhibition of cell growth, we measured cell viability with the aid of CellTiter-Glo luminescent cell viability assay (Figure 2C). Data revealed that addition of 2DG and/or metformin inhibited cell viability in a dose dependent manner in all EWS cells tested. At 2.5 mM of 2DG this inhibition was significant for all the cells. Metformin at 5 mM, in combination with 2DG induced profound inhibition for all the cell lines. Since, CellTiter-Glo uses ATP generated by metabolically active cells as a read out for cell viability, we further confirmed the results using an image cytometer (Celigo), where direct cell numbers were quantified. (Figure 2D). Cells were treated with either 5 mM 2DG or 10 mM metformin, or a combination of both. The results again demonstrated the inhibitory effect of both 2DG and metformin when cells were directly counted. We further confirmed our findings by evaluating the effect of the two drugs on a patient derived tumor xenograft (PDX) cell line PDX38, which was established in our lab. The tumor was derived from a patient with localized ES. Our data from CellTiter-Glo assay showed that both 2DG and metformin alone could effectively inhibit the growth of this PDX-derived cell line (Figure 2E). Overall, results from additional cell lines (Supplementary Figure 1) show that other than the exception of one cell line (CHLA-258), all EWS cells tested were sensitive to 2DG alone, or to the combination with metformin as demonstrated by significant reduction in cell viability. Compared to the malignant cells, when nonmalignant cells were treated with 5 mM 2DG, both cell lines specifically showed resistance to 2DG up to 5 mM for 72 hours treatment (Figure 2F).

### 2DG and metformin mediated inhibition of EWS cells persists under hypoxia and low glucose condition

ES tumors can typically display hypoxic areas within the center of the rapidly growing tumor mass [48, 49]. We investigated the ability of metabolic modulators to inhibit EWS cell growth under hypoxia and low glucose conditions. Contrary to previous reports [44], we found that metformin was effective under hypoxia, as well as under normoxia towards exerting its inhibitory effect (Figure 2G). Similar effects were seen when cells were grown in glucose free media, as significant inhibitory effect was seen in cell viability for all three EWS cells tested. The level of inhibition observed with 5 mM metformin in regular culture media containing 15-25 mM glucose (Figure 2H) was further augmented when cells were exposed to metformin under glucose free conditions in all three cell lines (*p* = 0.0001 for A673 and TC71 while *p* = 0.0057 for MHH).

### 2DG alone or in combination with metformin can induce apoptosis in EWS cells

**Figure 3:**
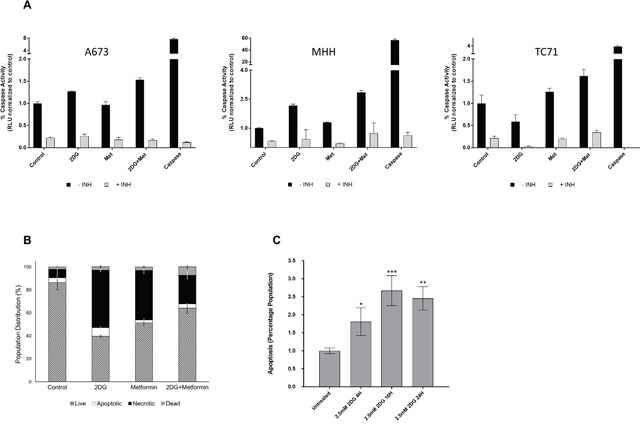
Metabolic inhibition induces apoptosis **(A)** Caspase-3 activity was measured using lysates from untreated cells or cells treated with 2DG and/or metformin as described under Materials and Methods. Purified caspase-3 was used as positive control. Results show fold change over corresponding untreated control. **(B)** Flow cytometry analysis showing percentage of cells undergoing apoptosis and necrosis in untreated, 2DG (2.5 mM), metformin (5 mM) or metformin and 2DG treated cells. Cells stained only with Propidium iodide (PI) were gated as dead cells and Annexin V-positive cells were gated as apoptotic cells. Necrotic cells were quantified as both Annexin V and PI positive population, using a FACS analyzer. **(C)** Flow cytometry analysis showing induction of apoptosis in a time dependent manner when treated with 2.5 mM 2DG in MHH cells. Data shown are means ± SEM (n = 3).

After determining that 2DG and metformin can inhibit EWS cell viability, we were interested in investigating whether the reductions in cell numbers as observed in Figure 2C and 2D were secondary to apoptosis. Measurement of caspase-3 activity (Figure 3A) showed that in the two out of the three cell lines, 2DG was successful in inducing a strong caspase-3 activity compared to untreated control. Although metformin could only induce a modest level of activity, the combination of the two drugs had significant effect as seen by high caspase-3 activity. In accordance to this observation, when cells were treated with 2DG and/or metformin in the presence of a pan caspase inhibitor (Z-VAD-FMK), or specific caspase-3 inhibitor, (Z-DEVD-FMK), the inhibition on cell viability was at least partially relieved (Supplementary Figure 2). Flow cytometry analysis using a GFP-Certified^™^ Apoptosis/Necrosis detection kit with A673 cells further confirmed this data (Figure 3B) with cells treated for 24 hours with 2.5 mM 2DG alone or in combination with 5 mM metformin. By 72 hours this proceeded to necrosis (data not shown). Since 2DG seemed to induce the highest level of apoptosis, we further examined the time dependent induction under the treatment of 2.5 mM 2DG and results show significant increase in apoptotic population from 4 hours onward, reaching a peak at 16h, within a 24h period (Figure 3C).

To further examine the cell death mechanism, we looked at changes in mitochondrial membrane potential depolarization associated with events upstream of apoptosis. Flow cytometry analysis revealed a strong depolarization with both drugs (Supplementary Figure 3). After observing evidence of apoptosis, we performed initial molecular studies to determine if enhanced cellular stress was associated with this therapeutic regimen. We specifically focused on examining AMP-activated protein kinase-α (AMPK-α), a metabolic master switch and energy sensor that is activated in low energy states [50, 51]. AMPK-α activation leads to inhibition of catabolic pathways and stimulation of anabolic pathways, thus directly affecting downstream targets including increasing the phosphorylation of Acetyl-CoA Carboxylase (ACC) [52]. Therefore, we assessed AMPK-α activation and downstream effectors via Western blotting (Supplementary Figure 4A). Both 2DG and metformin could upregulate AMPK-α activation by phosphorylation at threonine 172 residue and could inhibit ACC, as observed by an increase in ACC phosphorylation at serine 79 residue. Quantification of the bands showed that both 2DG and metformin induced AMPK-α phosphorylation at a modest level, but phosphorylation of ACC was significantly higher (Supplementary Figure 4B) than the control.

### Metabolic targeting inhibits cell proliferation and cell cycle progression in EWS cells

**Figure 4:**
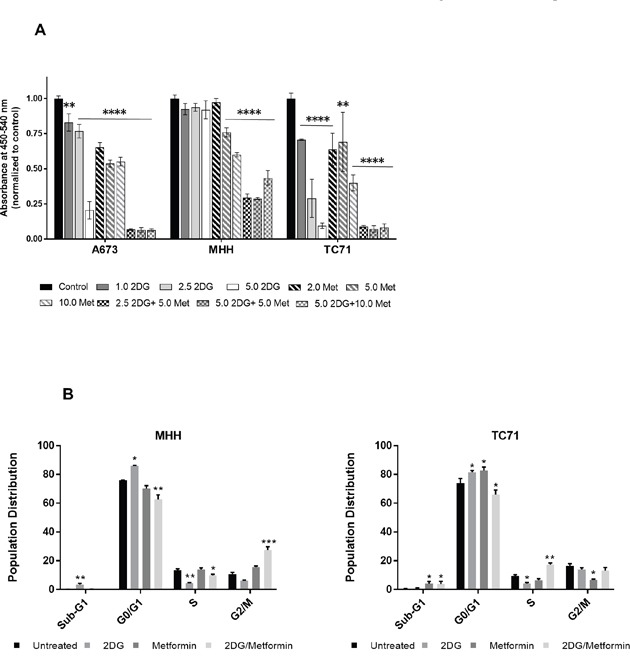
2DG and metformin can modulate cell proliferation and cell cycle **(A)** EWS cells, untreated or treated for 3 days with 2.5 mM 2DG, 5 mM metformin, or a combination of the two were quantified for proliferative cells by BrdU incorporation. **(B)** Cell cycle analysis by FACS of EWS cells treated with 2DG or metformin or a combination of both. Cells were treated for 72 h followed by staining with propidium iodide (PI). DNA content was quantified by flow cytometry. Analysis was done using FlowJo software with Dean-Jen Fox statistical model. Data is represented as the percentage of cells in the sub-G1, G0/G1, S and G2/M phase of the cell cycle. For each sample 10,000 cells were acquired. Data shown are means ± SEM. Pairwise comparisons of control to treated groups individually using Dunnett's multiple comparison test is shown in the figure (*p* = * <0.05, ** <0.01, *** <0.001, **** <0.0001).

In our experiments, we observed that although metformin treatment by itself did not induce apoptosis, the total number of cells remained much less than the untreated control. To account for this discrepancy, we looked at the effect of metformin and 2DG on cell proliferation by BrdU incorporation. Under the conditions used, we observed that, 2DG exerted little to modest effect at lower concentrations, while metformin, in a dose dependent manner, could induce dramatic inhibitory effect on cell proliferation (Figure 4A). Interestingly, although 2DG was not very effective in reducing the proliferation when acting alone, the combination with metformin had a more significant effect than either drug alone.

By modulating the cell's energy production with these two drugs, we expected that the cells would be energy deficient which would have an inhibitory effect on energy demanding processes such as DNA synthesis or cell cycle progression. Supporting this hypothesis, flow cytometry based cell cycle analysis with two different EWS cell lines, TC71 and MHH, revealed that 2DG increased the number of cells in G0/G1, while reducing it in S phase, thus inducing G1/S checkpoint arrest (Figure 4B).

### Metabolic inhibition significantly reduced stem-like cells in EWS

Cancer stem cells are highly tumorigenic and play a major role in metastasis and therapy resistance. Studies have reported that EWS contains an ALDH^high^ stem-like population that can confer resistance to chemotherapy [29]. In recent years, increased interest has been shown towards metabolic reprogramming specifically of the cancer stem cell population [53]. The role of metformin in targeting cancer stem cells has been evaluated in several studies and showed to elicit its anti-tumor effect by inducing bioenergetic stress on cancer stem cells [54–57]. Similarly, 2DG has been implicated in inhibiting self-renewal properties of cancer stem cells by modulating cellular metabolism [38, 58].

**Figure 5:**
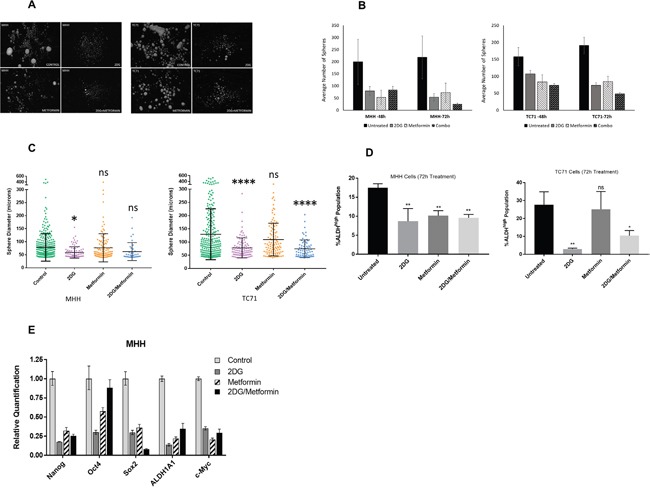
2DG and metformin can modulate Ewing sarcoma stem cell subpopulation cells **(A)** Sphere formation assay of MHH and TC71 cells. Sphere-forming ability of cells were greatly reduced in cells treated for 72 hours with 2.5 mM 2DG, or 5 mM metformin, or a combination of both, compared to untreated control. **(B)** Quantification of spheres determined with the help of Image ProPremiere (Media Cybernetics) software. Values represent the mean ± SD of triplicate. **(C)** Differences in diameter of spheres left untreated or treated for 72 hours with 2.5 mM 2DG, or 5 mM metformin, or a combination of both. Pairwise comparisons of control to treated groups individually using Dunnett's multiple comparison test is shown in the figure (*p* * <0.05, ** <0.01, *** <0.001, **** <0.0001). **(D)** Graphical representation of flow cytometry analysis using an Aldefluor assay kit showing ALDH^(high)^ activity. Experiment was set up according to the manufacturer's protocol as described in Materials and Methods. DEAB was used as an inhibitor of ALDH^(high)^ activity. Data shown are means ± SEM (n = 3). **(E)** Gene expression profile for stem cell markers. Cells were treated for 3 days with 2.5 mM 2DG, or 5 mM metformin, or a combination of both. Gene expression profile representing stem cell markers was done from extracted RNA and compared to untreated control.

To test whether 2DG and metformin can target putative cancer stem cells in EWS, we first evaluated the effect on sarcosphere formation using either drug alone or in combination (Figure 5). Sarcosphere forming assay is a surrogate for self-renewal capacity in tumor cells. We observed that 2DG and metformin significantly reduced sphere number (Figure 5A & 5B) while 2DG was more effective in reducing the size (Figure 5C) of the spheres when compared to untreated cells. Metformin alone had a less profound effect, and the drug combination seemed to derive most of its effect from 2DG. To further explore the effect of metabolic inhibition on the stem cell population we assayed the change in ALDH^high^ population in cells treated with either 2DG or metformin alone or in combination (Figure 5D). Like our previous observations, we found the most significant effect in the cancer stem cell compartment was with 2DG (2.5 mM) treatment for 72 hours. Additionally, to examine the effect of 2DG and metformin on cancer stem-like cells, we quantified the expression of genes associated with “stemness” including *OCT-4*, *Nanog, Sox-2*, *ALDH1A*, and *c-Myc*. Analysis of qPCR data in EWS cells showed that the expression level for all the genes went down significantly when treated with 2DG or metformin alone, or in combination (Figure 5E).

### Pretreatment of EWS cells with 2DG delays tumor growth and decreases metastatic load

**Figure 6:**
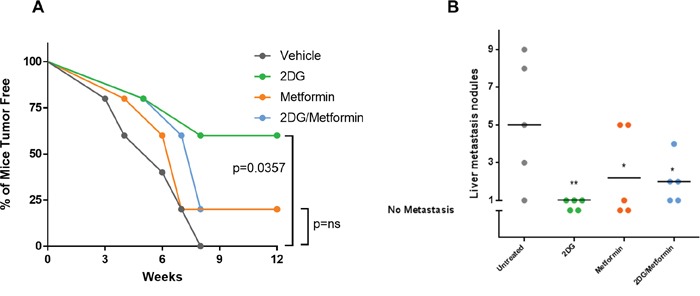
Pre-treatment with 2DG alone or in combination with metformin can delay tumor latency Untreated cells or cells pre-treated with either 2DG (2.5 mM), metformin (5 mM), or combination for 72 hours were harvested and 50,000 cells were injected in the left tibia of 5 mice in each group. **(A)** Graph showing tumor latency in mice. Each dot represents one mouse reporting a palpable tumor at the site of injection at indicated time points. **(B)** Graph showing number of metastatic nodules in liver from each mouse estimated by gross observation. Each dot represents one mouse with number of nodules in the Y-axis. No tumor or no metastasis indicates mice that had either no palpable tumor or no liver metastasis respectively. All statistical comparison was done pair-wise by comparing control group to each of the treated group using one-way Anova, with no multiple correction. NS indicates no significant difference, while *p* <0.05 was considered statistically significant and indicated with a *, and *p* = <0.01 with **.

Our *in vitro* results prompted us to evaluate the effect of 2DG and metformin in tumor initiation, using an orthotopic, xenograft mouse model. We pretreated EWS cells for 72 hours with 2DG (2.5 mM) or metformin (5 mM) alone, or a combination of the two prior to injecting 50,000 viable cells into the tibia of immunocompromised mice as described in materials and methods. Bi-weekly tumor volume measurement for 10-12 weeks showed a significant increase in tumor latency when cells were pretreated with 2DG (Figure 6A). Metformin treatment did show a trend toward delayed tumor formation, but this did not reach significance. The combination of 2DG and metformin had a similar, though slightly delayed, outcome as the metformin alone group. Both metformin alone and 2DG/metformin appeared inferior to 2DG alone in delaying and preventing tumor formation. Total metastatic burden was measured in terms of number of nodules, and gross weight of metastatic liver nodules, which revealed a significant reduction in tumor burden from all 2DG pretreated cells (Figure 6B; Supplementary Figure 5). Although metformin alone, or in combination with 2DG could significantly reduce the number of metastatic liver nodules (Figure 6A), in terms of weight this effect was antagonized (Supplementary Figure 5). Overall, the most significant reduction was observed in tumors generated from 2DG pretreated EWS cells.

### 2DG can enhance sensitivity to chemotherapy and targeted agents

**Figure 7:**
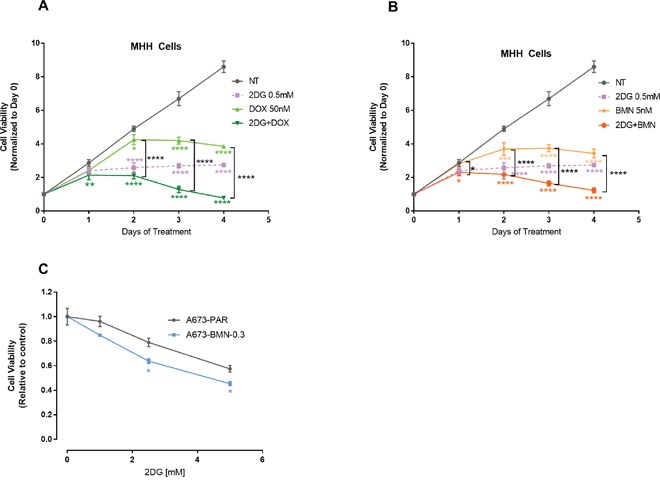
2DG can enhance chemosensitivity MHH cells were treated for 0-96 hours and viability was measured at indicated time points. **(A)** Cells were either untreated or treated with 2DG or Doxorubicin alone or in combination. **(B)** Cells were either left untreated or treated with 2DG or BMN alone or in combination. Data was normalized to cell viability reading from day 0. **(C)** PARP-i resistant cells are sensitive to 2DG mediated inhibition. Parental (A673) and the PARP inhibitor, BMN 673 (Talazoparib) resistant (A673-BMN) A673 cells were treated for 3 days with varying concentrations of 2DG. CellTiter-Glo was added and viability was measured at 72 hours. The results are expressed as relative fraction viability compared with the corresponding untreated control group. Data shown are means ± SD of 4 determinations. Pairwise comparisons using one-way Anova with Dunnett's multiple comparison test is shown in the figure (*p* * <0.05, ** <0.01, *** <0.001, **** <0.0001). Comparison of control to treated groups individually are indicated in asterisk with corresponding color while comparison between two treated groups are shown in black.

EWS-FLI1, the translocation product in EWS cells has been reported to directly bind to PARP-1, and regulate its expression. Thus, EWS-FLI1 has been proposed to be a bio-marker for PARP-inhibitor (PARP-i) sensitivity [18, 59, 60]. In spite of that, clinical trials have failed to show significant sensitivity to PARP-i. Along with this, development of chemo-resistance is common in EWS. To address this issue, we first examined if treatment with 2DG can add to the efficacy of a routinely used chemotherapeutic drug Doxorubicin and also to a PARP inhibitor, Talazoparib (BMN-673). We observed that 2DG can significantly increase the inhibitory effect of Doxorubicin (Figure 7A) and BMN-673 (Figure 7B). In both cases, significance was reached within 2 days of treatment. In order to explore the effect of metabolic inhibition as a potential means to overcome PARP-i resistance in EWS cell lines, we developed in our lab A673 cells resistant to Talazoparib (BMN-673), up to 300 nM, which is 50 times the IC_50_ value for the parental cells. When treated with increasing concentrations of 2DG, the resistant cell line showed more sensitivity when compared to the parental cell line (Figure 7C). Metformin did not seem to have any significant effect on reducing cell viability (data not shown).

## DISCUSSION

In recent years, the hallmarks that drive or characterize tumor growth have been revisited to include reprogramming of energy metabolism [32]. This feature was first described by Otto Warburg almost a century ago [30], who hypothesized that in most tumor cells, energy is generated from transformation of glucose to lactate, or aerobic glycolysis, where as in normal cells, pyruvate oxidation in mitochondria provides the energy. This renewed interest in tumor cell metabolism has led to several studies exploring the therapeutic potential of targeting glycolysis either alone or in combination with inhibitors of mitochondrial respiration. Studies done in breast, prostate, pancreatic, and esophageal cancer, have shown inhibition of tumorigenic properties and general tumor growth using this strategy [61–64].

Cancer cell specific metabolic adaptations have been targeted strategically in several pre-clinical and clinical studies (reviewed in [65, 66]). Many metabolic alterations involve upregulation of pro-tumorigenic factors, such as insulin and elevated level of glucose. Thus, anti-diabetic drugs like metformin has been a rational choice for some of these trials. But even with promising pre-clinical results with metformin, data from clinical trials have been confounding and inconsistent. Similarly, therapeutic potential of 2DG against cancer has been explored for a long time as well. In one of the early studies involving several types of cancer, side effects akin to mild hypoglycemia was observed that subsided within 90 minutes after infusion [67]. This effect was much less with oral administration of 2DG. Unfortunately, at this dose, no observable anti-tumor effect was noted and due to the risk associated with higher doses, the trial was discontinued. However, it is becoming evident that in combination with other treatment regimens, there is a significant therapeutic window for 2DG. Combination of 2DG and radiation in phase I/II clinical trials have shown at well tolerated levels of 2DG combined with radiotherapy, an increase in survival [68]. Similarly, trials using a combination with other drugs, such as docetaxel in patients with solid tumor [69], resulted in partial response in an aggressive breast cancer and disease progression was prevented for 8 weeks in some other patients. A relatively recent paper investigated sensitivity of several sarcoma cell lines to 2DG with or without metformin and found that osteosarcoma and embryonic rhabdomyosarcoma (eRMS) to be partially or relatively insensitive to 2DG. But when a combination of 2DG and metformin was used, the cells were sensitized enough to be significantly inhibited [65]. Thus, effective targeting of two separate yet related pathways can be developed into a viable therapeutic intervention.

While preparing this manuscript, a study was published demonstrating the efficacy of metabolic inhibition of energy pathways in EWS cells through NAMPT, the rate limiting enzyme of NAD [70]. It is encouraging that multiple approaches to inducing energy stress through metabolic inhibition can indeed be a specific and effective therapeutic strategy. To our knowledge, our study is the first one to show sensitivity of EWS cells to metabolic inhibition both directly as well as through affecting tumor stem-like cells as demonstrated by our *in vivo* data. EWS display at least three features which, make them attractive targets for this type of therapy: 1) basal glucose uptake and dependence upon glycolysis to meet primary energy needs, 2) high LDH activity and corresponding high levels of intracellular lactate, and 3) decreased mitochondrial functionality [65]. Our results show, compared to the two non-malignant cell types, EWS cells do have high lactate production indicating the fact that they rely uniquely on the use of aerobic glycolysis for their growth even in the presence of sufficient oxygen. To measure sensitivity to metabolic inhibitors we initially characterized the bioenergetic profiles of the EWS cells using Seahorse^™^. The EWS cells demonstrated much higher respiration rate and glycolytic reserve than the NM cells. Interestingly one of the EWS cell lines, namely A673 demonstrated more dependence on glycolysis than on mitochondrial respiration, when compared to the other two cell lines, MHH and TC71. Thus, to test our hypothesis that metabolic interference with glucose based inhibitors of glycolysis may represent a potentially effective therapy for ES, we used 2DG, a glycolysis inhibitor. In addition, we used metformin as the inhibitor of mitochondrial respiration.

While 2DG was shown to be more effective, a combination of the two agents showed mild to moderate additive effect in all the cell lines. We also noted that the differences in the bioenergetics profile of the EWS cells did not correlate to their sensitivity to 2DG, which indicates there might be other underlying characteristics involved. In our *in vitro* studies, we found metformin to have moderate effects compared to a stronger inhibitory effect of 2DG, when both agents where used alone. Furthermore, we observed that, 2DG had a more cytotoxic effect while, metformin had a more cytostatic effect. Our results indicate EWS cells are significantly sensitive to 2DG. In certain conditions we see an additive effect of metformin in combination with 2DG, though in other conditions a neutral or antagonistic effect is seen when compared to 2DG alone. The apparent antagonism is most notable in the *in vivo* experiments where 2DG's antineoplastic effect appears blunted when combined with metformin (Figure 6A). An increase in metformin's dose from 5 mM to 10 mM seems to revert this antagonism *in vitro*. This apparently confounding result is in fact not out of the ordinary. The *in vitro* doses required to see metformin's anti-tumor activity have been reported at much higher levels than clinically achievable (sometimes 100x). This could be due to the differences in cell culture conditions where glucose concentrations are at hyperglycemic levels. Studies have also shown tissue accumulation of metformin to be much higher from plasma concentrations and different concentrations are required for different modes of operation. In hepatic cells, it has been reported that high concentration of metformin is required to inhibit complex I [71]. Curiously enough, one cell line, namely CHLA-258 was resistant to 2DG but dramatically sensitive to metformin. Further metabolic characterization needs to be done to effectively address these apparent contradictions.

Targeting exclusively the bulk of the tumor cells might not always be an effective therapy model due to the presence of stem like cells. This subpopulation with their uncontrolled self-renewal and pluripotency have been determined to have increased tumorigenic and metastatic potential [72]. We observed that treatment of 2DG was very effective in significantly reducing tumor stem-like cells, as indicated by the multiple assays. The sphere-formation assay showed not only a reduction in the number of spheres but in their sizes as well. Treated EWS cells analyzed for standard embryonic and cancer stem cell markers, *Nanog*, *Oct-4, Sox-2*, *ALDH1A*, and *c-myc* [73, 74], showed significant reduction in these genes, indicating a decrease in cancer stem-like cell population. Treatment with 2DG was also very effective in reducing the subpopulation with high ALDH^high^ expression, previously shown to be associated with chemotherapy resistant EWS stem cells [29].

We attempted to address one of the major hurdles of current therapy models, the development of chemo-resistance. Our data indicates that 2DG can further potentiate conventional chemotherapy drugs such as Doxorubicin. Further, as mentioned earlier, 2DG treatment was effective against the subpopulation of cells that might be contributing to chemo-resistance. 2DG was clearly effective against a cell line that was strongly resistant to PARP inhibition by exhibiting an increase in 2DG sensitivity compared to the parental cell line. Interestingly, the PARP-i resistant cells showed minimal sensitivity to metformin alone. The combination of the two metabolic modulators did not diminish cell viability beyond the effects of 2DG alone. Further characterizations need to be performed to determine if there are any genetic, epigenetic or metabolic alterations associated with the PARP-i resistance underlying this observation.

As eluded in the introduction, a significant concern for EWS patients is the high rates of disease recurrence, leading to an extremely poor prognosis. Targeting EWS metabolism holds promise as a future treatment strategy, however further study is needed in determining the best agents and strategy for achieving this. A promising therapeutic role for 2DG in particular could lie in their use to enhance the antitumor effect of cytotoxic chemotherapy or as part of a maintenance therapy regimen. Our *in vivo* data, showed that cells pretreated with 2DG for only three days had a significant delay in tumor growth. Thus, we provide evidence that treatment with 2DG, following a standard treatment protocol could combat residual stem-like EWS cells and prevent disease recurrence. Overall, our work suggests that the clinical development of metabolic modulators may provide important therapeutic tools against this devastating disease; however, it also highlights the complicated nature of tumor metabolism.

## MATERIALS AND METHODS

### Cell culture

Human Ewing Sarcoma cell lines were procured from ATCC. A673 were grown in Dulbecco's Modified Eagle's Medium (DMEM); MHH and TC71 were grown in RPMI medium. The nonmalignant cell lines used, namely, IMR-90, are lung fibroblastic cells and hFOBs, are of osteoblastic lineage. IMR-90 were grown in Eagle's Minimum Essential Medium, and hFOBs were grown in 1:1 mixture of Ham's F12 Medium with Dulbecco's Modified Eagle's Medium, supplemented with 2.5 mM L-glutamine (without phenol red), and 0.3 mg/ml G418. All media were supplemented with 10% FBS. Cells were grown in a humidified atmosphere containing 5% CO2 at 37°C.

### Reagents and antibodies

2DG (D6134), Metformin (#D150959-5G), Valinomycin (V0627-10MG), and Staurosporine (89157-676) were purchased from Sigma-Aldrich. Primary antibodies p-Thr172-AMPK-α (#2535), AMPK-α (#2603), p-Ser79-ACC (#3661), ACC (#3676) were procured from Cell Signaling Technology. Antibody against β-Actin (NB600-503) was procured from Novus Biologicals. Secondary antibodies to mouse (Anti-mouse IgG, HRP-linked Antibody #7076) and to rabbit (Anti-rabbit IgG, HRP-linked Antibody 7074) were purchased from Cell Signaling Technology.

### Cell viability assay

Two different assays were used to measure cell viability. First, CellTiter-Glo Luminescent Cell Viability Assay (Promega) was used to measure ATP generated by metabolically active cells as an indicator of cell viability. Second, in a non-ATP dependent manner, the number of cells were assessed from a 96-well plate using an *in situ* image cytometer (Celigo, Nexcelom Bioscience, Lawrence, MA). Assays were done using the manufacturer's protocol. Briefly, 1-2 × 10^4^ cells per well were cultured in 96-well plates in the absence or presence of the drugs as indicated. Treatment was done for 72 hours unless otherwise indicated. At the end of the treatment, 100 μl of CellTiter-Glo reagent was added. Luminescence was recorded in a Multiskan FC (ThermoFisher) plate reader luminometer with an integration time of 1 s per well. All wells in both assays were done in triplicates.

### Caspase-3 activity assay

Caspase-3 activity was measured using Caspase 3 Assay Kit (CASP3C-1KT) from Sigma-Aldrich, according to the manufacturer's protocol. Briefly, cells (treated or control) were trypsinized after indicated duration of treatment and then suspended in 1X lysis buffer at the concentration of 10^6^ cells/100 μL. Cells were then incubated on ice with intermittent vortexing and finally cleared lysates were collected. For the assay, 5 μL cell lysates or caspase 3 positive control, with or without inhibitor were plated in 96-well plates along with assay buffer and substrate as described in the protocol. Plates were incubated from 2 hours to overnight at 37°C and read at 405 nm using a Multiskan FC (ThermoFisher) plate reader.

### Cell cycle analysis

Standard protocols were followed using flow cytometry with Propidium Iodide Flow Cytometry Cell Kit (#ab139418) from Abcam. Cell cycle analysis was done by using FlowJo software.

### Brdu incorporation – cell proliferation assay

Cells were seeded in 96 well plates and either left untreated or treated with inhibitors for 72 hours as indicated. Cells were allowed to be labeled with BrdU for the last 24 hours of the treatment. A colorimetric BrdU incorporation immunoassay was used (EMD Millipore, catalog number QIA58) according manufacture's instruction. Labeling was measured using a spectrophotometric plate reader at dual wavelengths of 450-540 nm.

### Apoptosis and necrosis assay

To determine distribution of apoptotic versus necrotic cell population, the GFP-Certified^™^ Apoptosis/Necrosis detection kit for microscopy and flow cytometry from Enzo Life Sciences was used. Cells were either left untreated or treated with metabolic inhibitors (2DG, and/or metformin), or a positive inducer such as Staurosporine at indicated concentration and duration. Cells were then stained according to manufacturer's protocol and analyzed with a BD LSRFortessa flow cytometer using Cyanine-3 and 7-AAD filters.

### Mitochondrial membrane permeability assay

The membrane potential in the cells with or without treatment was measured using the cationic dye JC-1 (ENZ-52304, Enzo Life Sciences), which exhibits potential-dependent accumulation in mitochondria, indicated by a fluorescence emission shift from green (525 nm) as monomers to red (590 nm) as aggregates. Mitochondrial depolarization is indicated by a decrease in the red-to-green fluorescence-intensity ratio using BD LSRFortessa flow cytometer. Valinomycin was used as a positive control as a potent depolarization inducer.

### Determination of lactate production

EWS cells (2×10 4 cells/well) were grown for 6, 12, and 24h in 6-well plates. Treatments were done only for 24h time points. The measurement of lactate was performed with a standard clinical samples method with internal standards as controls using YSI 2329 Lactate Membrane and an YSI 2700 Bioanalyzer.

### Metabolic profiling of cells using seahorse

Measurement of oxygen consumption rate (OCR) and extracellular acidification rate (ECAR) were done and analyzed using the XF24 Extracellular Flux Analyzer (Seahorse Bioscience). Briefly, 10-20,000 cells were seeded in each well and incubated overnight at 37 °C. Mitochondrial respiration were measured by taking sequential measurements of OCR from baseline to first injection of oligomycin to inhibit ATP synthase (1 μg/mL final concentration), followed by FCCP (2-5 μM) to uncouple mitochondrial oxidative phosphorylation, consequently to give maximal respiration reading, and finally injecting rotenone (1 μM) to inhibit mitochondrial complex I. For ECAR measurements, and in turn to compare the glycolytic profiles of the EWS cells with that of non-malignant cells, assays were carried out in glucose free media by plating cells as described before. For ECAR readings, initial baseline reading was measured first then real time changes in 3 minutes of increment was followed up by addition of first glucose (10 mM), followed by oligomycin (1 μg/mL), which inhibits mitochondrial ATP synthase enzyme, and lastly 2DG (2.5 mM), which inhibits glycolysis.

### Protein extraction and western blot analysis

Total cell extracts were prepared using RIPA buffer with protease inhibitors (Roche) with additional PMSF (1 mM), sodium orthovanadate (1 mM) and sodium fluoride (50 mM) added just before use. Protein concentration was quantified using the Pierce BCA colorimetric assay (ThermoFisher) and quantified against BSA standards with a Multiskan plate imager at 550 nM. Extracted proteins were resolved on 4-15% TGX gels (Bio-Rad) and transferred to PVDF membranes using iBlot dry transfer system (Invitrogen). Immunoblots were developed with the help of indicated primary antibodies and corresponding HRP tagged secondary antibodies). Blots were scanned using the ThermoFisher myECL imager.

### Relative quantification of gene expression

Total RNA was harvested using Trizol extraction method. The qScript cDNA SuperMix (QuantaBio, Beverly, MA, USA) was used to synthesize cDNA from 200ng of total RNA from each sample. Relative quantification of mRNA expression of genes were examined by quantitative RT-PCR (qRT-PCR) with iTaq Universal SYBR Green supermix (Bio-Rad). Reactions were performed on a StepOnePlus^™^ System. All reactions were run in triplicate. Melting curve analysis verified that all primers yielded a single PCR product. Gene expressions were normalized to 18S to yield a 2^−ΔΔCt^ value. All primers were purchased from Sigma-Aldrich. Primer sequences are included in Supplementary Table 1.

### Aldeflour assay and fluorescence-activated cell sorting (FACS)

Stem-like cells in established EWS cell lines using the Aldefluor® assay kit (Stem Cell Technologies, Vancouver, BC) according to the manufacturer's instructions. To identify ALDH^high^ and ALDH^low^ cells, 1×10^6^ tumor cells were incubated with or without DEAB at 37°C for 30 minutes, sorted by flow cytometry (LSRFortessa, BD Biosciences, Franklin Lakes, NJ) and analyzed using FACSDiva software. Dead cells were excluded from the analysis using DAPI. Individualized DEAB samples for each cell line and different treatment conditions were used to select the brightest and the dimmest ALDH-expressing tumor cells.

### Sphere-formation assay

To assess stem cell like growth that is independent of anchorage, sphere-formation assay was performed. Cells at 80% confluence were dissociated into single cell suspension and trypsin-EDTA was removed by washing with PBS. Cells were then seeded onto an ultra-low attachment 6-well plate (Corning) after counting cells and diluting to a concentration of 2×10^4^ cells/well in DMEM-F12 media (phenol red free), supplemented with B27, human recombinant epidermal growth factor (EGF; 20 ng/ml), and basic fibroblast growth factor (bFGF; 20 ng/ml). Cells were cultured up to 72 hours and imaged every 24 hours. The size and shape of the spheres were assessed using the Image ProPremiere (Media Cybernetics) software suite.

### *In vivo* xenograft model

MHH cells were cultured as described above. Tumor cells at 70% confluence were either left untreated or treated for 72 hours with 2DG (2.5 mM), metformin (5 mM) or both. Tumor cells were then collected and washed. Cell suspension with 50,000 viable tumor cells mixed with matrigel (1:1) were injected into the right tibia of 5-6 weeks old NSG mice (The Jackson Laboratory). Mice were housed in a pathogen-free environment and checked twice a week for tumor growth. Mice were sacrificed either at maximum allowable tumor burden or after 12 weeks, whichever was earlier. Primary bone tumors were dissected, weighed and harvested for additional studies. Selective animal necropsy of the lungs and the liver was performed to evaluate the presence of metastatic disease. Metastatic liver tumors were also dissected, weighed and harvested. All procedures were conducted per IACUC approved protocol at Baylor College of Medicine animal facility.

## SUPPLEMENTARY MATERIALS FIGURES AND TABLES



## References

[1] Riggi N, Stamenkovic I (2007). The biology of Ewing sarcoma. Cancer Lett.

[2] Lau YS, Adamopoulos IE, Sabokbar A, Giele H, Gibbons CL, Athanasou NA (2007). Cellular and humoral mechanisms of osteoclast formation in Ewing's sarcoma. Br J Cancer.

[3] Balamuth NJ, Womer RB (2010). Ewing's sarcoma. Lancet Oncol.

[4] Karski EE, Matthay KK, Neuhaus JM, Goldsby RE, Dubois SG (2013). Characteristics and outcomes of patients with Ewing sarcoma over 40 years of age at diagnosis. Cancer Epidemiol.

[5] Esiashvili N, Goodman M, Marcus RB (2008). Changes in incidence and survival of Ewing sarcoma patients over the past 3 decades: surveillance epidemiology and end results data. J Pediatr Hemat Oncol.

[6] Jiang Y, Ludwig J, Janku F (2015). Targeted therapies for advanced Ewing sarcoma family of tumors. Cancer Treat Rev.

[7] Gaspar N, Hawkins DS, Dirksen U, Lewis IJ, Ferrari S, Le Deley MC, Kovar H, Grimer R, Whelan J, Claude L, Delattre O, Paulussen M, Picci P (2015). Ewing sarcoma: current management and future approaches through collaboration. J Clin Oncology.

[8] May WA, Lessnick SL, Braun BS, Klemsz M, Lewis BC, Lunsford LB, Hromas R, Denny CT (1993). The Ewing's sarcoma EWS/FLI-1 fusion gene encodes a more potent transcriptional activator and is a more powerful transforming gene than FLI-1. Mol Cell Biol.

[9] Beauchamp E, Bulut G, Abaan O, Chen K, Merchant A, Matsui W, Endo Y, Rubin JS, Toretsky J, Uren A (2009). GLI1 is a direct transcriptional target of EWS-FLI1 oncoprotein. J Biol Chem.

[10] Wakahara K, Ohno T, Kimura M, Masuda T, Nozawa S, Dohjima T, Yamamoto T, Nagano A, Kawai G, Matsuhashi A, Saitoh M, Takigami I, Okano Y, Shimizu K (2008). EWS-Fli1 up-regulates expression of the Aurora A and Aurora B kinases. Mol Cancer Res.

[11] Feng FY, Brenner JC, Hussain M, Chinnaiyan AM (2014). Molecular pathways: targeting ETS gene fusions in cancer. Clin Cancer Res.

[12] Kelleher FC, Thomas DM (2012). Molecular pathogenesis and targeted therapeutics in Ewing sarcoma/primitive neuroectodermal tumours. Clin Sarcoma Res.

[13] DuBois SG, Marina N, Glade-Bender J (2010). Angiogenesis and vascular targeting in Ewing sarcoma: a review of preclinical and clinical data. Cancer.

[14] Wagner L, Turpin B, Nagarajan R, Weiss B, Cripe T, Geller J (2013). Pilot study of vincristine, oral irinotecan, and temozolomide (VOIT regimen) combined with bevacizumab in pediatric patients with recurrent solid tumors or brain tumors. Pediatr Blood Cancer.

[15] Chao J, Budd GT, Chu P, Frankel P, Garcia D, Junqueira M, Loera S, Somlo G, Sato J, Chow WA (2010). Phase II clinical trial of imatinib mesylate in therapy of KIT and/or PDGFRalpha-expressing Ewing sarcoma family of tumors and desmoplastic small round cell tumors. Anticancer Res.

[16] Gorlick R, Janeway K, Lessnick S, Randall RL, Marina N, Committee COGBT (2013). Children's Oncology Group's 2013 blueprint for research: bone tumors. Pediatr Blood Cancer.

[17] Jimeno A, Daw NC, Amador ML, Cusatis G, Kulesza P, Krailo M, Ingle AM, Blaney SM, Adamson P, M; Hidalgo, Children's Oncology Group (2007). Analysis of biologic surrogate markers from a Children's Oncology Group Phase I trial of gefitinib in pediatric patients with solid tumors. Pediatr Blood Cancer.

[18] Brenner JC, Feng FY, Han S, Patel S, Goyal SV, Bou-Maroun LM, Liu M, Lonigro R, Prensner JR, Tomlins SA, Chinnaiyan AM (2012). PARP-1 inhibition as a targeted strategy to treat Ewing's sarcoma. Cancer Res.

[19] Vormoor B, Curtin NJ (2014). Poly(ADP-ribose) polymerase inhibitors in Ewing sarcoma. Curr Opin Oncol.

[20] Grier HE, Krailo MD, Tarbell NJ, Link MP, Fryer CJ, Pritchard DJ, Gebhardt MC, Dickman PS, Perlman EJ, Meyers PA, Donaldson SS, Moore S, Rausen AR (2003). Addition of ifosfamide and etoposide to standard chemotherapy for Ewing's sarcoma and primitive neuroectodermal tumor of bone. New Engl J Med.

[21] Suresh R, Ali S, Ahmad A, Philip PA, Sarkar FH (2016). The role of cancer stem cells in recurrent and drug-resistant lung cancer. Adv Exp Med Biol.

[22] Doherty MR, Smigiel JM, Junk DJ, Jackson MW (2016). Cancer stem cell plasticity drives therapeutic resistance. Cancers.

[23] Abbott A (2006). Cancer: the root of the problem. Nature.

[24] Ginestier C, Hur MH, Charafe-Jauffret E, Monville F, Dutcher J, Brown M, Jacquemier J, Viens P, Kleer CG, Liu S, Schott A, Hayes D, Birnbaum D (2007). ALDH1 is a marker of normal and malignant human mammary stem cells and a predictor of poor clinical outcome. Cell Stem Cell.

[25] Rasper M, Schafer A, Piontek G, Teufel J, Brockhoff G, Ringel F, Heindl S, Zimmer C, Schlegel J (2010). Aldehyde dehydrogenase 1 positive glioblastoma cells show brain tumor stem cell capacity. Neuro Oncol.

[26] Tirino V, Desiderio V, Paino F, De Rosa A, Papaccio F, La Noce M, Laino L, De Francesco F, Papaccio G (2013). Cancer stem cells in solid tumors: an overview and new approaches for their isolation and characterization. FASEB J.

[27] van den Hoogen C, van der Horst G, Cheung H, Buijs JT, Lippitt JM, Guzman-Ramirez N, Hamdy FC, Eaton CL, Thalmann GN, Cecchini MG, Pelger RC, van der Pluijm G (2010). High aldehyde dehydrogenase activity identifies tumor-initiating and metastasis-initiating cells in human prostate cancer. Cancer Res.

[28] Liu G, Yuan X, Zeng Z, Tunici P, Ng H, Abdulkadir IR, Lu L, Irvin D, Black KL, Yu JS (2006). Analysis of gene expression and chemoresistance of CD133+ cancer stem cells in glioblastoma. Mol Cancer.

[29] Awad O, Yustein JT, Shah P, Gul N, Katuri V, O'Neill A, Kong Y, Brown ML, Toretsky JA, Loeb DM (2010). High ALDH activity identifies chemotherapy-resistant Ewing's sarcoma stem cells that retain sensitivity to EWS-FLI1 inhibition. PloS One.

[30] Warburg O, Wind F, Negelein E (1927). The metabolism of tumors in the body. J Gen Physiol.

[31] Gillies RJ, Robey I, Gatenby RA (2008). Causes and consequences of increased glucose metabolism of cancers. J Nucl Med.

[32] Hanahan D, Weinberg RA (2011). Hallmarks of cancer: the next generation. Cell.

[33] Sanchez-Sanchez AM, Antolin I, Puente-Moncada N, Suarez S, Gomez-Lobo M, Rodriguez C, Martin V (2015). Melatonin cytotoxicity is associated to warburg effect inhibition in Ewing sarcoma cells. PloS One.

[34] Wick AN, Drury DR, Nakada HI, Wolfe JB (1957). Localization of the primary metabolic block produced by 2-deoxyglucose. J Biol Chem.

[35] Kang HT, Hwang ES (2006). 2-Deoxyglucose: an anticancer and antiviral therapeutic, but not any more a low glucose mimetic. Life Sci.

[36] Stein M, Lin H, Jeyamohan C, Dvorzhinski D, Gounder M, Bray K, Eddy S, Goodin S, White E, Dipaola RS (2010). Targeting tumor metabolism with 2-deoxyglucose in patients with castrate-resistant prostate cancer and advanced malignancies. Prostate.

[37] Pelicano H, Martin DS, Xu RH, Huang P (2006). Glycolysis inhibition for anticancer treatment. Oncogene.

[38] Ciavardelli D, Rossi C, Barcaroli D, Volpe S, Consalvo A, Zucchelli M, De Cola A, Scavo E, Carollo R, D'Agostino D, Forli F, D'Aguanno S, Todaro M (2014). Breast cancer stem cells rely on fermentative glycolysis and are sensitive to 2-deoxyglucose treatment. Cell Death Dis.

[39] Dowling RJ, Niraula S, Stambolic V, Goodwin PJ (2012). Metformin in cancer: translational challenges. J Mol Endocrinol.

[40] Pollak MN (2012). Investigating metformin for cancer prevention and treatment: the end of the beginning. Cancer Discov.

[41] Foretz M, Guigas B, Bertrand L, Pollak M, Viollet B (2014). Metformin: from mechanisms of action to therapies. Cell Metab.

[42] Pernicova I, Korbonits M (2014). Metformin--mode of action and clinical implications for diabetes and cancer. Nat Rev Endocrinol.

[43] Camacho L, Dasgupta A, Jiralerspong S (2015). Metformin in breast cancer - an evolving mystery. Breast Cancer Res.

[44] Garofalo C, Capristo M, Manara MC, Mancarella C, Landuzzi L, Belfiore A, Lollini PL, Picci P, Scotlandi K (2013). Metformin as an adjuvant drug against pediatric sarcomas: hypoxia limits therapeutic effects of the drug. PloS One.

[45] Owen MR, Doran E, Halestrap AP (2000). Evidence that metformin exerts its anti-diabetic effects through inhibition of complex 1 of the mitochondrial respiratory chain. Biochem J.

[46] Andrzejewski S, Gravel SP, Pollak M, St-Pierre J (2014). Metformin directly acts on mitochondria to alter cellular bioenergetics. Cancer Metab.

[47] Wu M, Neilson A, Swift AL, Moran R, Tamagnine J, Parslow D, Armistead S, Lemire K, Orrell J, Teich J, Chomicz S, Ferrick DA (2007). Multiparameter metabolic analysis reveals a close link between attenuated mitochondrial bioenergetic function and enhanced glycolysis dependency in human tumor cells. Am J Physiol Cell Physiol.

[48] Dunst J, Ahrens S, Paulussen M, Burdach S, Jurgens H (2001). Prognostic impact of tumor perfusion in MR-imaging studies in Ewing tumors. Strahlenther Onkol.

[49] Knowles HJ, Schaefer KL, Dirksen U, Athanasou NA (2010). Hypoxia and hypoglycaemia in Ewing's sarcoma and osteosarcoma: regulation and phenotypic effects of Hypoxia-Inducible Factor. BMC Cancer.

[50] Hardie DG, Carling D (1997). The AMP-activated protein kinase--fuel gauge of the mammalian cell?. Eur J Biochem.

[51] Carling D (2017). AMPK signalling in health and disease. Curr Opin Cell Biol.

[52] Hardie DG, Pan DA (2002). Regulation of fatty acid synthesis and oxidation by the AMP-activated protein kinase. Biochem Soc Trans.

[53] Menendez JA, Joven J, Cufi S, Corominas-Faja B, Oliveras-Ferraros C, Cuyas E, Martin-Castillo B, Lopez-Bonet E, Alarcon T, Vazquez-Martin A (2013). The Warburg effect version 2.0: metabolic reprogramming of cancer stem cells. Cell Cycle.

[54] Rattan R, Ali Fehmi R, Munkarah A (2012). Metformin: an emerging new therapeutic option for targeting cancer stem cells and metastasis. J Oncol.

[55] Bednar F, Simeone DM (2012). Metformin and cancer stem cells: old drug, new targets. Cancer Prev Res.

[56] Hirsch HA, Iliopoulos D, Tsichlis PN, Struhl K (2009). Metformin selectively targets cancer stem cells, and acts together with chemotherapy to block tumor growth and prolong remission. Cancer Res.

[57] Hirsch HA, Iliopoulos D, Struhl K (2013). Metformin inhibits the inflammatory response associated with cellular transformation and cancer stem cell growth. Proc Nat Acad Sci U S A.

[58] Shen YA, Wang CY, Hsieh YT, Chen YJ, Wei YH (2015). Metabolic reprogramming orchestrates cancer stem cell properties in nasopharyngeal carcinoma. Cell Cycle.

[59] Gill SJ, Travers J, Pshenichnaya I, Kogera FA, Barthorpe S, Mironenko T, Richardson L, Benes CH, Stratton MR, McDermott U, Jackson SP, Garnett MJ (2015). Combinations of PARP inhibitors with temozolomide drive PARP1 trapping and apoptosis in Ewing's sarcoma. PLoS One.

[60] Garnett MJ, Edelman EJ, Heidorn SJ, Greenman CD, Dastur A, Lau KW, Greninger P, Thompson IR, Luo X, Soares J, Liu Q, Iorio F, Surdez D (2012). Systematic identification of genomic markers of drug sensitivity in cancer cells. Nature.

[61] Ben Sahra I, Laurent K, Giuliano S, Larbret F, Ponzio G, Gounon P, Le Marchand-Brustel Y, Giorgetti-Peraldi S, Cormont M, Bertolotto C, Deckert M, Auberger P, Tanti JF, Bost F (2010). Targeting cancer cell metabolism: the combination of metformin and 2-deoxyglucose induces p53-dependent apoptosis in prostate cancer cells. Cancer Res.

[62] Cheng G, Zielonka J, Dranka BP, McAllister D, Mackinnon AC, Joseph J, Kalyanaraman B (2012). Mitochondria-targeted drugs synergize with 2-deoxyglucose to trigger breast cancer cell death. Cancer Res.

[63] Cheong JH, Park ES, Liang J, Dennison JB, Tsavachidou D, Nguyen-Charles C, Wa Cheng K, Hall H, Zhang D, Lu Y, Ravoori M, Kundra V, Ajani J (2011). Dual inhibition of tumor energy pathway by 2-deoxyglucose and metformin is effective against a broad spectrum of preclinical cancer models. Mol Cancer Ther.

[64] Cheng G, Zielonka J, McAllister D, Tsai S, Dwinell MB, Kalyanaraman B (2014). Profiling and targeting of cellular bioenergetics: inhibition of pancreatic cancer cell proliferation. Br J Cancer.

[65] Issaq SH, Teicher BA, Monks A (2014). Bioenergetic properties of human sarcoma cells help define sensitivity to metabolic inhibitors. Cell Cycle.

[66] Porporato PE, Dhup S, Dadhich RK, Copetti T, Sonveaux P (2011). Anticancer targets in the glycolytic metabolism of tumors: a comprehensive review. Front Pharmacol.

[67] Vander Heiden MG (2011). Targeting cancer metabolism: a therapeutic window opens. Nat Rev Drug Discov.

[68] Dwarakanath BS, Singh D, Banerji AK, Sarin R, Venkataramana NK, Jalali R, Vishwanath PN, Mohanti BK, Tripathi RP, Kalia VK, Jain V (2009). Clinical studies for improving radiotherapy with 2-deoxy-D-glucose: present status and future prospects. J Cancer Res Ther.

[69] Raez LE, Papadopoulos K, Ricart AD, Chiorean EG, Dipaola RS, Stein MN, Rocha Lima CM, Schlesselman JJ, Tolba K, Langmuir VK, Kroll S, Jung DT, Kurtoglu M (2013). A phase I dose-escalation trial of 2-deoxy-D-glucose alone or combined with docetaxel in patients with advanced solid tumors. Cancer Chemother Pharmacol.

[70] Mutz CN, Schwentner R, Aryee DN, Bouchard ED, Mejia EM, Hatch GM, Kauer MO, Katschnig AM, Ban J, Garten A, Alonso J, Banerji V, Kovar H (2017). EWS-FLI1 confers exquisite sensitivity to NAMPT inhibition in Ewing sarcoma cells. Oncotarget.

[71] He L, Wondisford FE (2015). Metformin action: concentrations matter. Cell Metab.

[72] Lee AS, Tang C, Rao MS, Weissman IL, Wu JC (2013). Tumorigenicity as a clinical hurdle for pluripotent stem cell therapies. Nat Med.

[73] Liu A, Yu X, Liu S (2013). Pluripotency transcription factors and cancer stem cells: small genes make a big difference. Chin J Cancer.

[74] Medema JP (2013). Cancer stem cells: the challenges ahead. Nat Cell Biol.

