# Supplementary material for: Metabolic modulation of Ewing sarcoma cells inhibits tumor growth and stem cell properties
Source: Oncotarget. 2017 Aug 24;8(44):77292–308. doi: 10.18632/oncotarget.20467 (PMC5652780; doi:10.18632/oncotarget.20467)
Supplement: Supplementary file 1 [file oncotarget-08-77292-s001.pdf]

Metabolic modulation of Ewing sarcoma cells inhibits tumor growth and stem cell properties

SUPPLEMENTARY MATERIALS

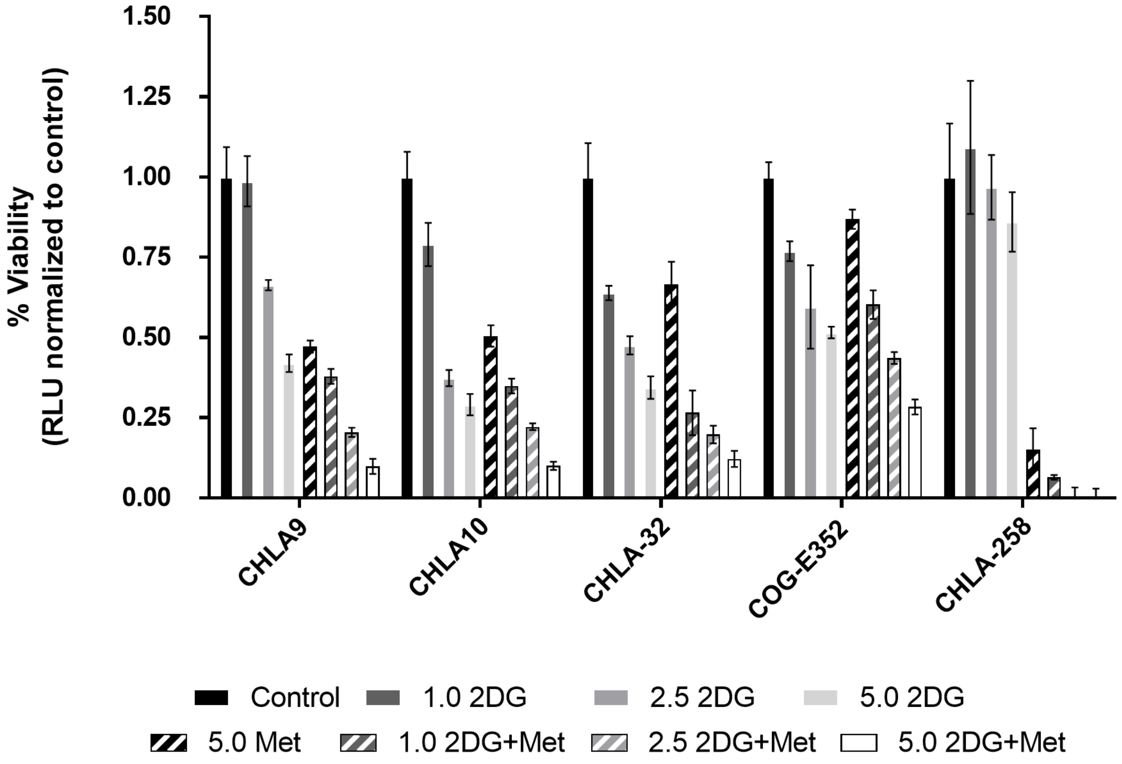

Supplementary Figure 1: Effect of 2DG and metformin on additional EWS cells. Cells were treated for 3 days with indicated concentrations of 2DG or metformin, alone or in combination. CellTiter-Glo was added and viability was measured at 72 hours. The results are expressed as relative fraction of viability compared with the corresponding untreated control group. Other than the indicated non-significant statistical difference (ns) all other treatment groups compared to the corresponding control was significantly different with p <0.0001.

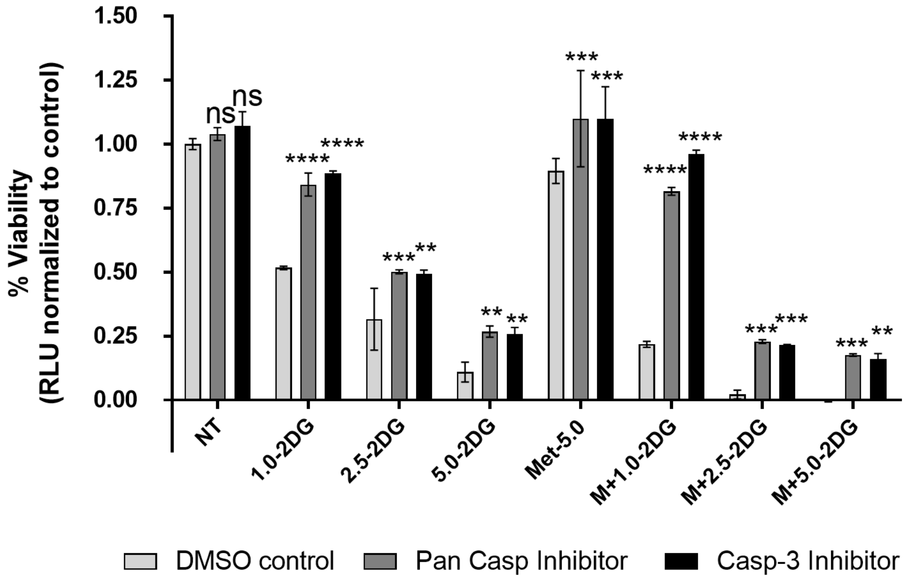

Supplementary Figure 2: Effect of caspase-3 inhibitor (10 µM Z-DEVD-FMK) and pan caspase inhibitor (10 µM Z-VAD-FMK) on 2DG and metformin treatment in EWS cells. Cells were treated for 3 days with indicated concentrations of 2DG or metformin, with or without the indicated inhibitors. CellTiter-Glo was added and viability was measured at 72 hours. The results are expressed as relative fraction of viability compared with the corresponding untreated control group.

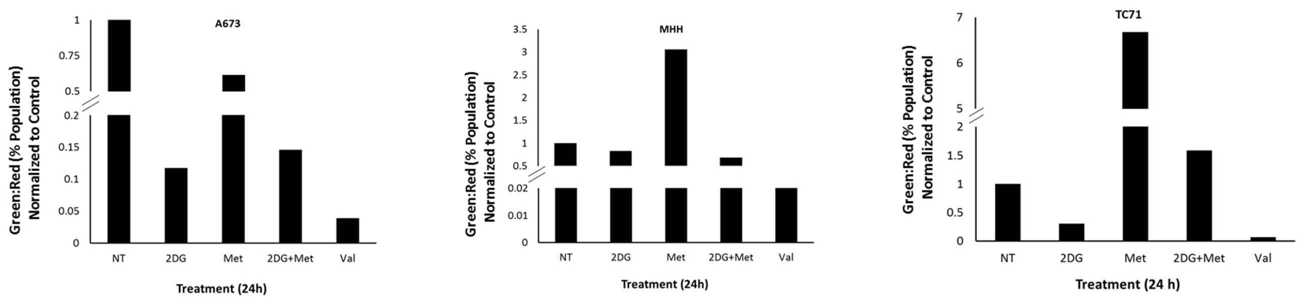

Supplementary Figure 3: Mitochondrial membrane potential (MMP) was analyzed by JC-1 fluorescence. Cells after 24 hours of treatment with 2DG, or metformin or a combination of both was analyzed with FACS. Data is represented as Green/Red ratio.

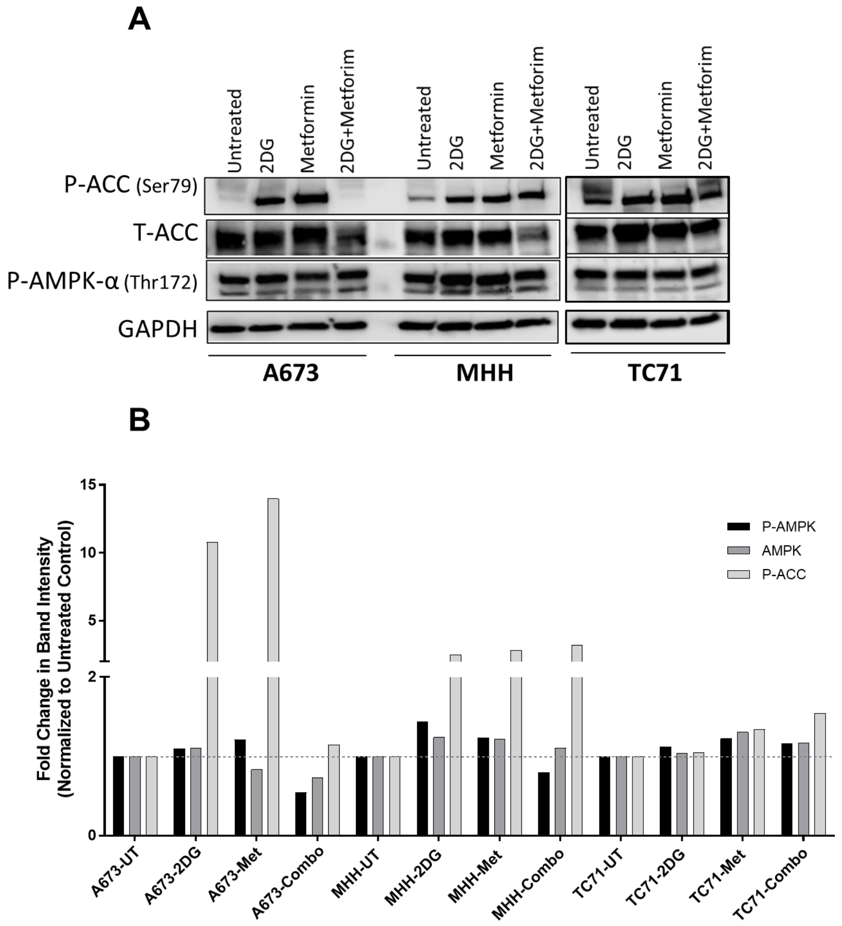

Supplementary Figure 4: Cellular energy sensing pathways are activated by 2DG and metformin. **(A)** Western blot analysis was done to investigate effect of 2DG and metformin on AMPK-α activation and downstream targets. EWS cell lines were either left untreated or treated with 2.5 mM 2DG or 5 mM metformin, or a combination of both for 48 hours and the levels of phosphorylated and total ACC, and phosphorylated AMPK-α were determined. GAPDH was used as the loading control. **(B)** Quantification of bands relative to untreated control expressed after normalized to GAPDH as the loading control.

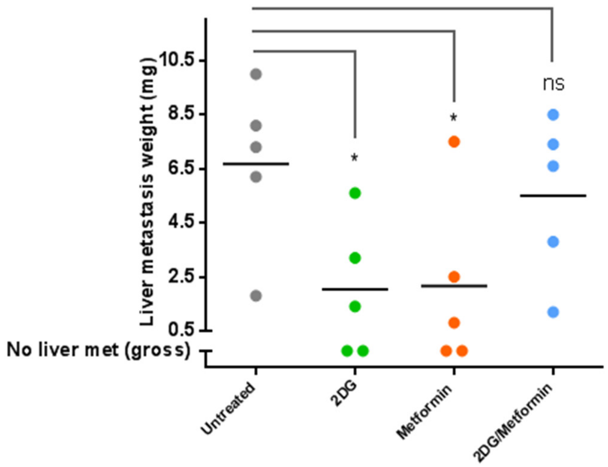

Supplementary Figure 5: Aggregate weight of liver metastatic tumor in each mouse. Each dot represents aggregate liver metastatic weight from one mouse. All statistical comparison is done pair-wise by comparing control group to each of the treated group using one-way Anova, with no multiple correction. *p* <0.05 was considered statistically significant and indicated with a star, while ns indicates no significant difference.

Supplementary Table 1: Primer sequences of genes analyzed with qRT-PCR

Gene	Forward primer	Reverse primer
18S	5' -GTAACCCGTTGAACCCCATT- 3'	5' -CCATCCAATCGGTAGTAGCG- 3'
ALDH1A	5’-CTGCTGGCGACAATGGAGT- 3’	5’-GTCAGCCCAACCTGCACAG-3’
c-Myc	5' -TCAAGAGGTGCCACGTCTCC- 3'	5' -TCTTGGCAGCAGGATAGTCCT- 3'
OCT-4	5' -GAGAACCGAGTGAGAGGCAACC- 3'	5' -CATAGTCGCTGCTTGATCGCTTG- 3'
Nanog	5' -AATACCTCAGCCTCCAGCAGATG- 3'	5' -TGCGTCACACCATTGCTATTCTTC- 3'
Sox2	5' -TGGACAGTTACGCGCACAT- 3'	5' -CGAGTAGGACATGCTGTAGGT- 3'
